# Use of Walnut Shell Powder to Inhibit Expression of Fe^2+^-Oxidizing Genes of *Acidithiobacillus Ferrooxidans*

**DOI:** 10.3390/ijerph13050461

**Published:** 2016-04-30

**Authors:** Yuhui Li, Yehao Liu, Huifang Tan, Yifeng Zhang, Mei Yue

**Affiliations:** 1 Department of Biological and Environmental Engineering, Hefei University, Hefei 230601, China; yuhuili112@163.com (Y.L.); 15805699883@139.com (H.T.); 13485672396@139.com (Y.Z.); 2 School of Public Health, Anhui Medical University, Hefei 230032, China; liuyehao@ahmu.edu.cn

**Keywords:** walnut shell powder, acid mine drainage, *Acidithiobacillus ferrooxidans*, *rus* operon

## Abstract

*Acidithiobacillus ferrooxidans* is a Gram-negative bacterium that obtains energy by oxidizing Fe^2+^ or reduced sulfur compounds. This bacterium contributes to the formation of acid mine drainage (AMD). This study determined whether walnut shell powder inhibits the growth of *A. ferrooxidans*. First, the effects of walnut shell powder on Fe^2+^ oxidization and H^+^ production were evaluated. Second, the chemical constituents of walnut shell were isolated to determine the active ingredient(s). Third, the expression of Fe^2+^-oxidizing genes and *rus* operon genes was investigated using real-time polymerase chain reaction. Finally, growth curves were plotted, and a bioleaching experiment was performed to confirm the active ingredient(s) in walnut shells. The results indicated that both walnut shell powder and the phenolic fraction exert high inhibitory effects on Fe^2+^ oxidation and H^+^ production by *A. ferrooxidans* cultured in standard 9K medium. The phenolic components exert their inhibitory effects by down-regulating the expression of Fe^2+^-oxidizing genes and *rus* operon genes, which significantly decreased the growth of *A. ferrooxidans*. This study revealed walnut shell powder to be a promising substance for controlling AMD.

## 1. Introduction

Acid mine drainage (AMD), which results from the oxidation of sulfur minerals by air and water, is a worldwide environmental problem [1]. It generates an extreme environment characterized by low pH and high concentrations of heavy metals [2]. AMD is mainly generated by the dissolution of sulfide ores and the production of ferric iron and H^+^ by certain acidophilic and chemolithotrophic bacteria. Fe^2+^-oxidizing, acidophilic, and chemolithotrophic bacteria, such as *Acidithiobacillus ferrooxidans*, are regarded the main producers in AMD environments [3,4]. The generated AMD can remain in the environment for a very long time, and AMD remediation is generally difficult and costly [5,6]. Hence, developing novel approaches for the efficient control of AMD production is imperative.

Currently, the optimal strategy for controlling AMD is the prevention of sulfide oxidation. Although many techniques have been developed to mitigate AMD, most have significant limitations. For example, several physical and chemical strategies that prevent sulfide oxidation are not cost effective and thus cannot be widely applied [7]. Studies have investigated the control of AMD generation through the sterilization of AMD-producing bacteria [2,8]. Although the use of bactericides is simple and economical, it is not a viable strategy because it entails repetitive usage for inhibiting bacterial populations. Furthermore, bactericides are toxic to aquatic organisms [9]. Therefore, developing a low-cost, sustainable, and environment-friendly method for controlling AMD is crucial.

The application of agricultural residue for the removal of toxic contaminants is a recent development in environmental technology [10]. The major advantage of this technology over conventional methods includes its low cost as well as its high efficiency, the possibility of metal recovery, and the broad range of operational conditions. Moreover, some types of agricultural residue inhibit bacterial growth [11,12]. Walnut shell not only contains antibacterial substances, but can also absorb heavy metal ions such as Ni and Mn [13,14]. We screened many types of agricultural residues in a previous experiment. Agricultural residues, including walnut shell, tea seed shell, and orange peels, inhibited AMD production. Among them, walnut shell showed the highest inhibition. Hence, in this study, the active compound of walnut shell was isolated, and the mechanism responsible for the inhibition of *A. ferrooxidans* growth was studied.

## 2. Materials and Methods

### 2.1. Determination of Walnut Shell Composition

Walnuts (*Juglandis hopeiensis* Hu) were purchased from a local supermarket. The lignin, cellulose, phenolic, and flavonoid components of walnut shell were extracted using previously described methods with some modifications. The phenolic component fraction was extracted using the method of Torun *et al.* [15,16] with some modifications. In brief, first, acetonitrile/acetic acid (15 mL, 96:4, *v*/*v*) was added to 10 g of walnut shell powder in a centrifuge tube. The mixture was stirred for 1 h in the dark and centrifuged at 12,000 g for 10 min at room temperature. Subsequently, the solids were extracted two more times by using 10 mL of the aforementioned extraction solvent; this mixture was shaken for 15 min in the dark and centrifuged under the aforementioned conditions. Finally, the phenolic portion was isolated from the extracts by using high polar dimer 600 (HPD600) macroporous resin. The concentration of phenolic compounds isolated from the extract was determined using the Folin–Ciocalteau method [15] and expressed in milligrams gallic acid equivalents per gram of dry matter by comparison with a calibration curve plotted using a pure standard compound.

To isolate flavonoids [17], 15 g of walnut shell powder was mixed with 150 mL of aqueous ethanol solution (70%, *v*/*v*), refluxed three times (2 h each time), filtered, and evaporated to a constant volume of 100 mL. Flavonoids were extracted from this liquid using AB-8 macroporous resin.

To extract lignin [18], 10 g of walnut shell powder was mixed with 100 mL of 6 M aqueous HCl and vigorously stirred (at room temperature) to obtain a pH of 2.0. After acidification, the precipitated lignin was centrifuged at 4000 rpm for 10 min, thoroughly washed with acidified water (pH 2.0), and oven-dried at 60 °C for 16 h until a constant mass was obtained, which was stored in a desiccator. Further purification by redissolving and precipitating was performed to remove extraneous organic contaminants and to reduce the low-molecular-weight fractions.

To obtain high-quality cellulose [19], 10 g of walnut shell powder was mixed with 100 mL of acetic-nitric acid mixture (80:20, *v*/*v*) at 120 °C for 20 min. Subsequently, the cellulose produced was rinsed with distilled water until neutral and oven-dried.

### 2.2. Strains, Medium, and Culture Conditions

The strain used in this experiment was *A. ferrooxidans* SY23, which was isolated from AMD in our previous research [20]. All experiments were performed in triplicate in 250-mL flasks containing 100 mL of solution at 30 °C on a shaker at 150 rpm (at an initial PH of 2.0). *A. ferrooxidans* was cultured in 9 K medium [21]. The phenolic component-cultured *A. ferrooxidans* cells were cultured in 9 K medium supplemented with the phenolic component extracted from walnut shell. *A. ferrooxidans* cultured in 9 K medium without any walnut shell extracts was used as the control.

### 2.3. Analytical Methods for the Culture

PH was monitored using a digital pH meter (model PHS-3C, Shanghai Precision & Scientific Instrument Co. Ltd., Shanghai, China). Fe^2+^ and total iron levels were measured using a colorimetric method with 1,10-phenanthroline through standard methods [22]. The Fe^3+^ level was calculated on the basis of the difference between total iron and Fe^2+^ levels.

### 2.4. Total RNA Extraction and Real-Time Polymerase Chain Reaction

Bacteria cultured in 9K medium in the presence or absence of the phenolic component were harvested through centrifugation. The harvest times for cells in the early logarithmic phase were determined based on the growth curves. Total RNA of *A. ferrooxidans* was extracted and purified using the RNApure bacteria kit (CWbio, Beijing, China). Total RNA was used as the template to synthesize cDNA by using the SuperQuickRT cDNA kit (CWbio, Beijing, China), according to the manufacturer’s instructions. The relative expression of genes was determined using real-time polymerase chain reaction (PCR) with the UltraSYBR Mixture kit (CWbio, Beijing, China). The sequences of primers in this experiment are detailed in previous studies [3,23,24]. The ratio of gene expression was recorded as the fold change in expression between the treated samples and the control. The results were normalized to the *ala*S gene which encodes alanyl tRNA synthetase. Real-time PCR experiments were performed in triplicate and used cDNA synthesized from RNA samples obtained from three independent cultures.

### 2.5. Growth Curves of A. ferrooxidans in 9K Medium Containing Different Components of Walnut Shell

A single source of *A. ferrooxidans* bacterial cells was resuspended in 9K medium, and suspensions containing 1.2 × 10^7^ cells·mL^−1^ were used as 10% (*v/v*) inoculum to ensure the same initial conditions. Bacterial cells were cultured in fresh 9K medium supplemented with walnut shell powder and its five isolated constituents (the concentrations used in this experiment were stated in results). Growth curves were plotted using the direct cell count determined in a Neubauer chamber.

### 2.6. Pyrite Bioleaching Solution Preparation and Bioleaching Experiment

A single source of *A. ferrooxidans* bacterial cells was resuspended in 0 K medium (containing 3.0 g (NH_4_)_2_SO_4_, 0.1 g KCl, 0.5 g K_2_HPO_4_, 0.5 g MgSO_4_·7H_2_O, and 0.01 g Ca(NO_3_)_2_ per 1000 mL H_2_O, pH 1.80), and suspensions containing 1.2 × 10^7^ cells·mL^−1^ were used as 10% (*v*/*v*) inoculum to ensure the same initial conditions. Three types of pyrite bioleaching solutions (PBSs) were prepared as described in Table 1 and were used in the experiments.

All bioleaching experiments were performed in 250-mL flasks; each flask contained 100 mL of one type of the aforementioned media and 1.0 g of pyrite. For culturing *A. ferrooxidans*, each flask was incubated with 10% (*v*/*v*) inoculum at 25 °C with shaking at 150 rpm. Leaching characteristics were monitored by determining the iron ion concentration and pH. The iron ion was measured using atomic absorption spectroscopy. The pH of the bioleaching solution was measured using a pH probe. Triplicate leaches were performed under identical conditions to ensure the reproducibility of the bioprocess experiments.

## 3. Results and Discussion

### 3.1. Walnut Shell Powder Inhibited the Oxidative Activity of A. ferrooxidans

**Figure 1:**
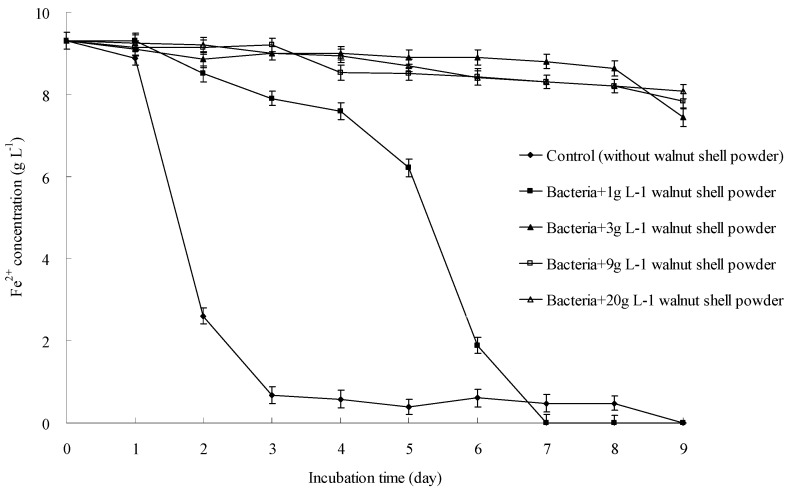
Inhibitory effect of different concentrations of walnut shell powder on Fe^2+^ oxidization.

**Figure 2:**
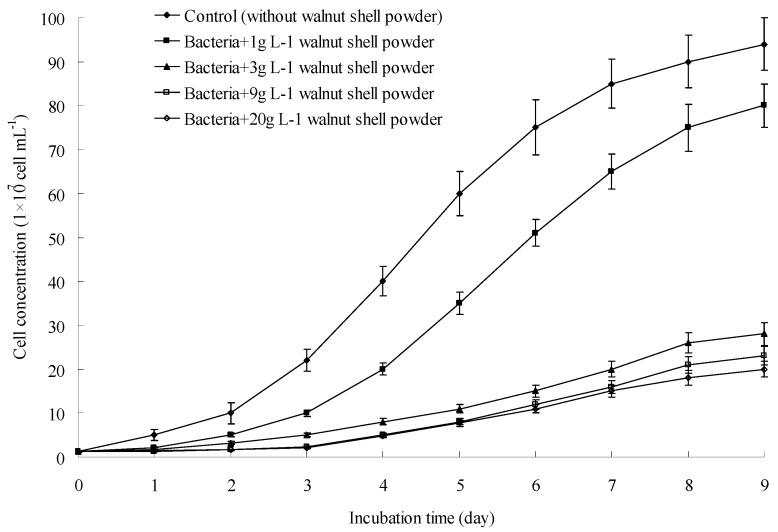
Growth curves of *A. ferrooxidans* with different concentrations of walnut shell powder.

As shown in Figure 1, after 3-day incubation of *A. ferrooxidans* SY23, the Fe^2+^ concentration in the control decreased rapidly from 9.3 g·L^−1^ to 0.7 g·L^−1^. The culture turned from light green to reddish brown. Growth curves were plotted to show the cell number during incubation (Figure 2). The curves showed that *A. ferrooxidans* multiplied rapidly, and Fe^2+^ was continuously oxidized into Fe^3+^. When 1 g·L^−1^ walnut shell powder was added to the culture, the rate of Fe^2+^ oxidation decreased from 9.3 g·L^−1^ to 7.6 g·L^−1^ during days 1–4. However, from day 4 to day 7, the Fe^2+^ concentration decreased rapidly from 7.6 g·L^−1^ to 0 after the addition of 1 g·L^−1^ walnut shell powder. This phenomenon was attributed to the mass of newly produced *A. ferrooxidans*. When the concentration of walnut shell powder was increased to 3, 9, and 20 g·L^−1^, the Fe^2+^ concentration only fluctuated between 8 and 10 g·L^−1^ and did not significantly change during the experimental period. This observation might be because the growth of *A. ferrooxidans* was significantly suppressed by high concentrations of walnut shell powder, and only some Fe^2+^ was oxidized to Fe^3+^. These data strongly support our hypothesis that walnut shell powder can suppress the growth of *A. ferrooxidans*. *A. ferrooxidans* experienced more stress when the concentration of walnut shell powder increased. The growth of *A. ferrooxidans* could be partly inhibited when the concentration of walnut shell powder was as low as 1g·L^−1^. Moreover, the growth of *A. ferrooxidans* stopped completely, and the Fe^2+^ concentration remained unchanged when the concentration of walnut shell powder increased to 3 g·L^−1^ or higher.

**Figure 3:**
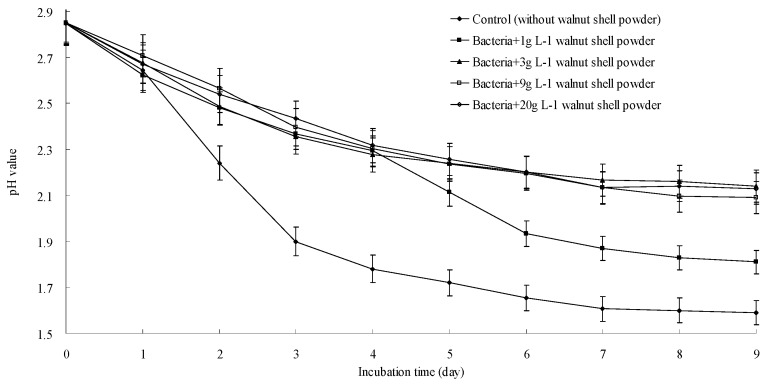
Inhibitory effect of different concentrations of walnut shell powder on H^+^ production.

As shown in Figure 3, the pH of the control showed a downward trend during the experimental period. Specifically, pH decreased rapidly from 2.8 to 1.9 within 3 days and stabilized at 1.6. However, when walnut shell powder was added at concentrations from 3 to 20 g·L^−1^, the pH of the cultures slowly decreased from 2.8 to 2.4 during the first 3 days and stabilized at approximately 2.1 during the experimental period. When walnut shell powder was added at 1 g·L^−1^, the final pH of the culture stabilized at 1.8, a value intermediate between the control and higher concentrations of walnut shell powder. These data indicated that the acidification caused by *A. ferrooxidans* was effectively alleviated by walnut shell powder, and the minimal concentration was 3 g·L^−1^.

### 3.2. Only the Phenolic Component Could Inhibit the Oxidative Activity of A. ferrooxidans

**Figure 4:**
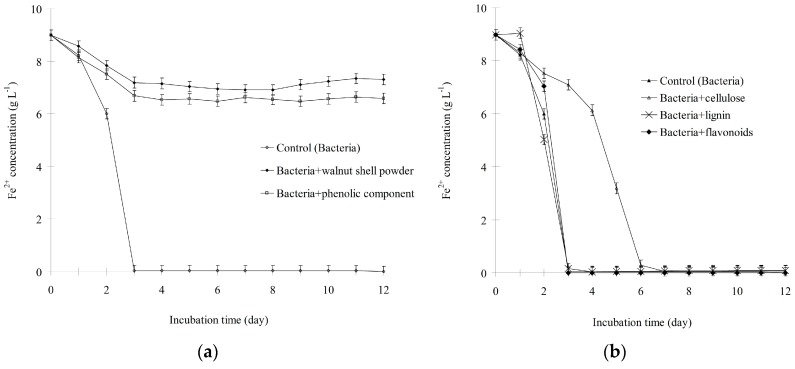
Inhibitory effect of walnut shell powder (**a**) and its isolated components (**b**) on Fe^2+^ oxidization.

**Figure 5:**
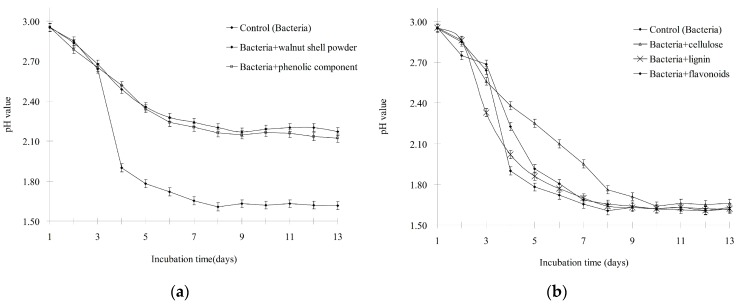
Inhibitory effect of walnut shell powder (**a**) and its isolated components (**b**) on H^+^ production.

To determine the active substance(s) in walnut shell, the components listed in Table 2 were isolated. Because the minimal concentration of walnut shell was 3 g·L^−1^, as mentioned earlier, the concentration of the compounds used in this experiment was equivalent to their abundances in 3 g of walnut shell. These compounds were added individually to cultures inoculated with *A. ferrooxidans*, and pH and the Fe^2+^ concentration were measured as described earlier. As shown in Figure 4, the Fe^2+^ concentration in the cultures treated with walnut shell powder or the phenolic fraction supplement slowly decreased from 9.0 to 6.6. By contrast, Fe^2+^ was completely oxidized in the control and cultures with the cellulose, lignin, and flavonoids supplements. These results indicated that the oxidative activity of *A. ferrooxidans* was inhibited only by walnut shell and the phenolic components. *A. ferrooxidans* can produce H^+^ by oxidizing Fe^2+^ and inorganic sulfur compounds. Therefore, we also tested whether the H^+^-producing ability of *A. ferrooxidans* is suppressed by compounds isolated from walnut shell. As shown in Figure 5, the pH of the sample treated with walnut shell powder or the phenolic component slowly decreased from 2.9 to 2.2. However, the pH of the control and samples treated with cellulose, lignin, and flavonoids decreased from 2.9 to 1.6. These data also supported that only the phenolic fraction of walnut shell could suppress the oxidizing activity of *A. ferrooxidans*.

Many studies have demonstrated the antibacterial activity of the phenolic component. Kchaou *et al.* evaluated the antimicrobial and cytotoxic activities of the phenolic fraction extracted from dates [25]. Similarly, Mahboubi *et al.* demonstrated the antibacterial activity of the phenolic components extracted from *Pleniflora* flowers [26]. Moreover, many findings have indicated that phenolics exhibit broad-spectrum antibacterial activity [11,12]. Our results also indicate that the phenolic components extracted from walnut shell show antibacterial activity against *A. ferrooxidans*.

### 3.3. Relative Expression of Fe^2+^-Oxidizing Genes Was Inhibited by the Phenolic Components

Because the Fe^2+^-oxidizing activity of *A. ferrooxidans* was inhibited significantly after treatment with the phenolic components extracted from walnut shell, it was deduced that the genes related to Fe^2+^ oxidation might be suppressed.

The fundamental genes involved in Fe^2+^ oxidation are a part of the *res* and *pet*I operons, and the Fe^2+^ oxidation regulators are encoded by *reg*BA, *cta*R, and *fur*. The results of real-time amplification are shown in Table 3. We found that the mRNA levels of Fe^2+^-oxidizing genes considerably decreased after treatment with the phenolic fraction. The Fe^2+^ oxidation regulators *reg*B and *res*B decreased by 1.8-fold at least and 14.7-fold when compared with the control.

Quantification of target gene expression provided insight into the regulation of the Fe^2+^-oxidizing system. The gene expression profiles of *A. ferrooxidans* showed that all Fe^2+^-oxidizing genes shared similar regulation mechanisms. The genes of the *res* and *pet*I operons were mainly regulated during bacterial growth, which is consistent with the role of these genes in electron transport, which is essential for growth in Fe^2+^-containing medium [27,28,29]. In our experiment, these genes were downregulated when the growth of *A. ferrooxidans* was delayed. The *reg*BA and *cta*R genes acted as inducers of the genes involved in the oxidation of Fe^2+^, such as those of the *res* and *pet*I operons. Kucera *et al.* demonstrated that the expression profiles of these genes share a similar pattern [3], which is consistent with our results.

### 3.4. Expression of Rus Operon Genes Was Suppressed by the Phenolic Fraction

The proteins of the *A. ferrooxidans* respiratory chain are encoded by *rus* operon genes, which are arranged in the *A. ferrooxidans* genome as follows: cyc2, cyc1, ORF1, coxB, coxA, coxC, coxD, and rus [23]. The *rus* operon is dependent on the energetic substrate Fe^2+^ or S^0^ [30]. Because an excess of unoxidized Fe^2+^ was present in the culture after treatment with the phenolic components, we hypothesize that the expression of *rus* operon genes was suppressed. To test the hypothesis, the expression of eight *rus* operon genes was measured. As shown in Table 4, all transcripts of the *rus* operon genes were more abundant in the control than in cells cultured with the phenolic fraction by 5.8-fold (cyc2) to 22.1-fold (*rus*). In comparison with the expression of other genes, the *rus* gene showed the most abundant expression.

Yarzabal *et al.* demonstrated that the *rus* operon is involved in Fe^2+^ oxidation rather than in S^0^ oxidation [23]. The expression of the *rus* operon is induced by ferrous Fe^2+^, and its transcript level rapidly increases during the active phase of Fe^2+^ oxidation and bacterial growth. However, our results indicated that in the presence of the phenolic components, the transcript levels of the genes of the *rus* operon decreased significantly, even at a high concentration of Fe^2+^. This finding could be attributed to the suppression of expression of *rus* operon genes by the phenolic component. Recent studies have revealed that phenolic compounds can regulate the gene expression of microbes, particularly Gram-negative bacteria. Li *et al.* found that the plant phenolic compound *p*-coumaric acid could repress gene expression in the *Dickeya dadantii* type III secretion system [31]. One phenolic compound, phenolic acid, had appreciable effects against indoleacetic acid production in *Pseudomonas fluorescens* and significantly suppressed gene expression of the type III secretion system [32]. Therefore, the aforementioned results are consistent with our findings that the phenolic components isolated from walnut shell could suppress the expression of some genes.

### 3.5. A. ferrooxidans Growth Could Be Inhibited by Walnut Shell Powder and the Phenolic Components

**Figure 6:**
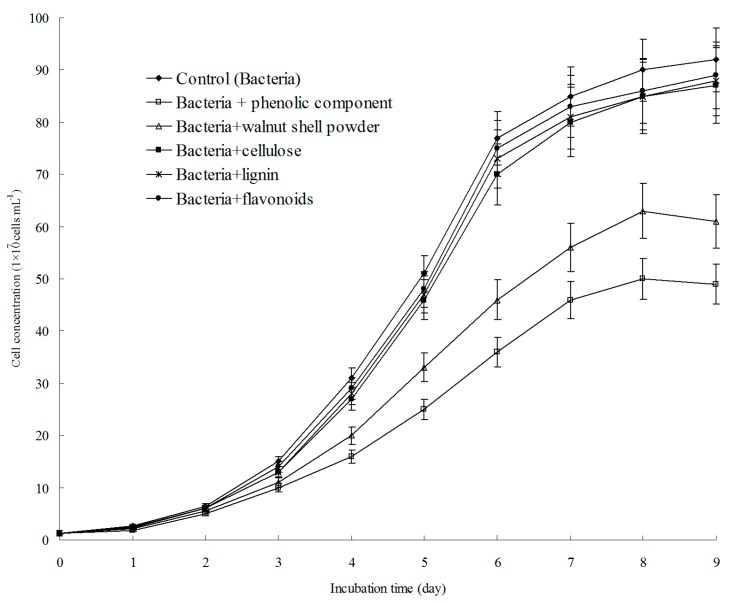
Growth curves of *A. ferrooxidans* with walnut shell powder and its five constituents.

As shown by the aforementioned findings, the phenolic fraction suppressed the oxidative activity and the expression of the *rus* operon of *A. ferrooxidans*. Hence, it was deduced that the growth of *A. ferrooxidans* could be delayed. As shown in Figure 6, the growth of *A. ferrooxidans* cultured in medium supplemented with walnut shell powder or the phenolic components was remarkably slower than the control, and the phenolics showed a higher inhibitory effect than walnut shell powder. Moreover, no difference was observed in the growth of *A. ferrooxidans* when the other three walnut shell constituents were added.

*A. ferrooxidans* obtains energy from Fe^2+^ or various reduced sulfur compounds. Some genes and the *rus* operon are critical for the survival of this bacterium [23,24]. In this study, we demonstrated that the growth rate of *A. ferrooxidans* decreased significantly when the expression of these critical genes was suppressed.

### 3.6. Result of Bioleaching Experiment Showed that the Phenolic Fraction Was the Active Ingredient

**Figure 7:**
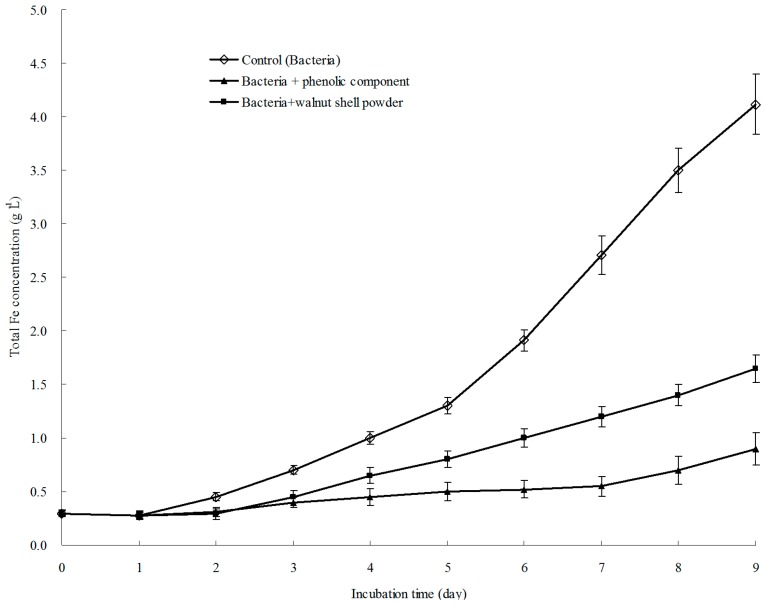
Effect on total Fe concentration after treatment with pyrite bioleaching solutions.

**Figure 8:**
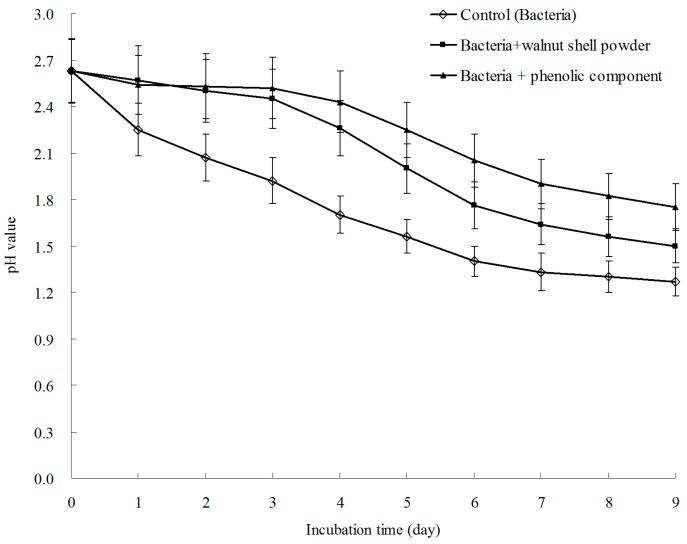
Effect on pH after treatment with pyrite bioleaching solutions.

Figure 7 and Figure 8 illustrate the total Fe and pH changes during the bioleaching experiment. *A. ferrooxidans* significantly promoted pyritic sulfur oxidation in the control, and the concentration of total Fe increased to 4.1 g·L^−1^ at the end of the experiment. Compared with the control, the oxidation of pyritic sulfur was suppressed in the presence of walnut shell powder and the phenolic components, and the concentrations of total Fe were 1.65 and 0.9, respectively. Moreover, the phenolic fraction showed higher activity than walnut shell powder. By contrast, the changes in the pH value showed a trend similar to that for with total Fe during the bioleaching experiment. The pH of the control rapidly decreased from 2.63 to 1.27. However, the addition of walnut shell powder or the phenolic fraction could slow down the pH value decrease.

Bioleaching, as an eco-friendly and energy-saving process, is widely used in metallurgical processing. It is also utilized for *in vitro* studies of *A. ferrooxidans* [33,34]. In our bioleaching experiment, walnut shell powder and its phenolic constituents showed high efficiency for inhibiting the oxidation of pyrite. By contrast, the isolated phenolic component exhibited higher inhibitory effects than walnut shell powder. These results support our aforementioned conclusions.

## 4. Conclusions

This study discovered a promising method for controlling AMD formation through the suppression of the *A. ferrooxidans* energy system. The phenolic fraction isolated from walnut shell effectively inhibited Fe^2+^ oxidation and H^+^ production by *A. ferrooxidans*. Moreover, the same result was obtained with walnut shell powder. The phenolic components downregulated the expression of Fe^2+^-oxidizing genes and the *rus* operon. Hence, they caused a decrease in Fe^2+^ oxidation and H^+^ production. Thus, walnut shell powder represents an eco-friendly and economical substance with promising applications in controlling AMD.

## Figures and Tables

**Table 1:** Pyrite bioleaching solutions used in this study.

Groups	Medium	The Walnut Shell Component Added
Control (Bacteria)	0 K + pyrite	None
Bacteria + walnut shell powder	0 K + pyrite	Walnut shell powder
Bacteria + phenolic fraction	0 K + pyrite	Phenolics

**Table 2:** Composition of walnut shell.

Component	Content (g·kg^−1^ Walnut Shell)	The Dosage Used in the Experiments (g)
Cellulose	350.7 ± 10.5	1.05
Lignin	314.7 ± 8.5	0.94
Phenolics	16.9 ± 1.9	0.051
Flavonoids	3.7 ± 0.5	0.011

Values are expressed as means ± standard deviations. The dosage of the compounds used in the experiment was equivalent to their abundances in 3 g of walnut shell.

**Table 3:** Quantification of Fe^2+^-oxidizing genes by real-time PCR.

Operon Name	Gene	Control	Phenolic Fraction	Ratio of Control to Total Phenolics
*res* operon	hyp	20.3 ± 2.4	7.5 ± 0.2	2.7
	resC	11.4 ± 1.3	4.1 ± 0.8	2.8
	resB	16.2 ± 2.1	1.1 ± 0.2	14.7
*pet*I operon	petC-1	7.2 ± 1.1	3.2 ± 0.4	2.3
	petB-1	16.2 ± 2.1	4.2 ± 0.2	3.9
	petA-1	26.2 ± 3.1	8.3 ± 0.6	3.2
	sdrA-1	17.9 ± 1.6	1.5 ± 0.1	11.9
	cycA-1	26.9 ± 3.2	12.1 ± 1.1	2.2
iron regulators	regA	6.2 ± 1.9	2.8 ± 0.7	2.2
	regB	30.8 ± 2.7	16.8 ± 1.9	1.8
	ctaR	17.2 ± 2.2	3.5 ± 0.4	4.9
	*fur*	5.4 ± 1.1	1.2 ± 0.3	4.5

All values are expressed as n-fold relative to the reference gene. Values shown are the mean of three independent experiments ± standard deviation.

**Table 4:** Quantification of *rus* transcripts by real-time PCR.

Gene	Control	Total Phenolics	Ratio of Control to Total Phenolics
*cyc*2	27.3 ± 2.2	4.7 ± 0.3	5.8
*orf*	80.1 ± 7.6	7.5 ± 0.2	10.7
*cyc*1	10.4 ± 0.9	0.6 ± 0.04	17.3
*cox*B	142.9 ± 8.3	8.7 ± 0.9	16.4
*cox*A	75.6 ± 6.6	8.1 ± 0.5	9.3
*cox*C	57.3 ± 4.3	9.4 ± 0.6	6.1
*cox*D	155.4 ± 7.3	13.6 ± 1.3	11.4
*rus*	227.5 ± 13.7	10.3 ± 0.5	22.1

All values are expressed as *n*-fold relative to the reference gene. Values shown are the mean of three independent experiments ± standard deviation.
